# Viral Hemorrhagic Septicemia Virus Activates Integrated Stress Response Pathway and Induces Stress Granules to Regulate Virus Replication

**DOI:** 10.3390/v15020466

**Published:** 2023-02-07

**Authors:** Barkha Ramnani, Shelby Powell, Adarsh G. Shetty, Praveen Manivannan, Brian R. Hibbard, Douglas W. Leaman, Krishnamurthy Malathi

**Affiliations:** 1 Department of Biological Sciences, University of Toledo, 2801 West Bancroft Street, Toledo, OH 43606, USA; 2 College of Sciences, Auburn University at Montgomery, 7400 East Dr., Montgomery, AL 36117, USA

**Keywords:** stress granule, VHSV, G3BP1, interferon, PERK, eIF2α phosphorylation, translation

## Abstract

Virus infection activates integrated stress response (ISR) and stress granule (SG) formation and viruses counteract by interfering with SG assembly, suggesting an important role in antiviral defense. The infection of fish cells by Viral Hemorrhagic Septicemia Virus (VHSV), activates the innate immune recognition pathway and the production of type I interferon (IFN). However, the mechanisms by which VHSV interacts with ISR pathway regulating SG formation is poorly understood. Here, we demonstrate that fish cells respond to heat shock, oxidative stress and VHSV infection by forming SG that localized key SG marker, Ras GTPase-activating protein (SH3 domain)-binding protein 1 (G3BP1). We show that PKR-like endoplasmic reticulum kinase (PERK), but not (dsRNA)-dependent protein kinase (PKR), is required for VHSV-induced SG formation. Furthermore, in VHSV Ia infected cells, PERK activity is required for IFN production, antiviral signaling and viral replication. SG formation required active virus replication as individual VHSV Ia proteins or inactive virus did not induce SG. Cells lacking G3BP1 produced increased IFN, antiviral genes and viral mRNA, however viral protein synthesis and viral titers were reduced. We show a critical role of the activation of ISR pathway and SG formation highlighting a novel role of G3BP1 in regulating VHSV protein translation and replication.

## 1. Introduction

Cells respond to stress stimuli like heat shock, amino acid deprivation, oxidative stress and virus infection, by activating the integrated stress response (ISR) pathway to restore cellular homeostasis [1,2]. Early response to stress involves shutting down protein translation to restrict the use of nutrients and energy by inhibiting translation initiation [3,4,5,6,7]. Virus infection triggers cellular stress and viruses have evolved strategies to counter host responses to sustain productive infection [8,9,10,11]. During virus infection, the accumulation of double-stranded RNA (dsRNA) replicative intermediates in the cytosol and viral proteins in the endoplasmic reticulum (ER) activates ISR through dsRNA-dependent protein kinase R (PKR) and PKR-like ER kinase (PERK) and eukaryotic translation initiation factor 2 alpha (eIF2α) phosphorylation causing translation repression [1,12,13,14,15,16,17,18]. The arrest of translation initiation and the disassembly of polysomes results in the accumulation of stalled mRNA initiation complexes and RNA-binding proteins in membraneless, phase-separated aggregates called stress granules (SG) [19,20,21,22,23,24,25].

SG are dynamic and assemble by aggregation initiated by RNA-binding proteins such as Ras-GTPase activating SH3 domain binding protein 1 (G3BP1), T cell internal antigen-1 (TIA-1) and other RNA-binding proteins driving a liquid-liquid phase separation through which stalled cytosolic mRNA along with translation factors are separated from the rest of the cytosol [26,27,28]. Following the resolution of stress, disassembly allows the sequestered mRNAs to return to translation to allow recovery [29,30]. Depending on the type of stress, the protein and RNA composition of SG varies and studies show specific transcripts required to combat stress are excluded from SG [21,31,32]. SG are considered signaling platforms and specific antiviral stress granules (avSG) formed during virus infection localize antiviral proteins like PKR, retinoic acid-inducible gene I (Rig-I), melanoma differentiation-associated protein 5 (MDA5), 2′-5′ oligoadenylate synthetase (OAS) and Ribonuclease L to provide a platform for interaction with RNA ligands to enhance type 1 interferon (IFN) production and antiviral defense [20,33,34,35]. The assembly of avSG is required for signaling to produce IFN during Newcastle Disease Virus (NDV), Influenza A Virus (IAV) and Sindbis Virus (SINV) infections [34,36,37]. During IAV infection, SG are induced and both IAV viral RNA and Rig-I are sequestered in SG, thereby providing a platform for sensing of viral RNA by Rig-I and cells lacking SG produced less IFN and higher viral titers [38]. In contrast, viruses like Encephalomyocarditis Virus (EMCV), Polio and Coxsackievirus B3 (CVB3) counteract SG formation by targeting SG proteins through G3BP1 cleavage by viral proteases or by inhibiting upstream eIF2α pathway to cause disassembly of SG and support viral replication [39,40]. G3BP1 facilitates replication of several viruses. For instance, Hepatitis C Virus (HCV) and Chikungunya Virus (CHIKV) use virus-encoded proteins to recruit G3BP1 to sites of replication and murine norovirus inhibits SG formation and redistributes G3BP1 to replication sites [41,42,43]. G3BP1 regulates the translation of norovirus genome by binding with VPg cap protein and directly recruiting ribosomes to enhance viral protein translation while host translation is shut off [44,45]. SGs are therefore considered an integral part of innate immune signaling pathways during viral infection.

Viral Hemorrhagic Septicemia Virus (VHSV), also known as Piscine novirhabdovirus, is one of the world’s most deadly infectious fish pathogens, infecting over 90 marine and freshwater species worldwide [46]. VHSV is a member of the Rhabdoviridae and more specifically the genus *Novirhabdovirus* based on the presence of a unique nonstructural gene, nonvirion (NV) [47]. VHSV is a bullet-shaped, enveloped virion containing a non-segmented, negative sense, single stranded RNA genome of approximately 11 kb that encodes six proteins in the order 3′-leader-N-P-M-G-NV-L-trailer-5′ [48]. The RNA genome codes for five structural proteins; nucleoprotein (N), phosphoprotein (P), matrix protein (M), glycoprotein (G), a RNA-dependent RNA polymerase (L) and a nonstructural protein (NV) [49]. Based on phylogenetic analyses of G and N genes, VHSV is classified into four genotypes (I–IV) [50]. Genotype I is further divided into five sub lineages (Ia to Ie), and Ia strain is virulent in rainbow trout and responsible for most outbreaks in European freshwater trout farms [50,51,52]. Genotype IV is further divided into three sub-lineages (IVa to IVc) and are virulent in several wild marine and freshwater fish, but not in rainbow trout. VHSV IVb was first reported in the Laurentian Great Lakes region in 2003 and infects only freshwater species [53,54,55].

The antiviral innate immune and IFN system is conserved among mammals and teleost fish [56,57,58]. In fish, similar to higher vertebrates, the viral nucleic acids are recognized by pattern recognition receptors like Rig-like helicases (Rig-I, MDA5) leading to signaling cascades and production of type I IFN and cytokines followed by induction of interferon-stimulated genes (ISGs) such as MX-1 with antiviral roles to clear infections [59,60,61,62]. The VHSV IVb infection of fish cells activates the host response pathway, however, the M protein of the VHSV IVb suppresses IFN production and antiviral responses at the transcriptional level [63]. The non-structural NV protein is essential for viral growth, replication and pathogenicity [64]. The deletion of IVb NV led to an earlier onset of viral-induced apoptosis and the interaction of NV with host antiviral proteins, proteins involved in stress response and IFNβ production, suggest a broader role in VHSV pathogenesis [64,65,66,67]. Our previous studies have shown that VHSV IVb infection in EPC cells regulates translation by activating PERK and inducing phosphorylation of eIF2α. NV protein is implicated in host translation shut-off and IFN signaling as NV-deficient recombinant VHSV IVb infection caused significantly lower phosphorylation of eIF2α and decreased viral protein synthesis [68]. These observations suggest that VHSV induces innate response by affecting the selective translation of viral and host proteins to regulate stress response pathway and SG formation. However, little is known about the molecular mechanisms underlying stress response and the role of SG in VHSV infection. Here, we show that VHSV infection induces SG formation in rainbow trout and EPC cells by activating ISR pathway. The activation of stress kinase PERK, and not PKR, is required for SG formation while both kinases are important for IFN signaling. SG formation required the replication of VHSV as inactive virus or the expression of the individual viral proteins did not induce SG formation. Using the CRISPR/Cas9 mediated knockout of G3BP1 in RTgill cells, we show that G3BP1 is required for VHSV replication and VHSV induced translation shut-off. Our results show an important role of activation of ISR pathway in SG formation and the critical role of G3BP1 in VHSV pathogenesis.

## 2. Materials and Methods

### 2.1. Cell Lines and Culture Conditions

Rainbow trout gonad (RTG2), rainbow trout gill (RTgill), Epithelioma papulosum cyprinid (EPC) and Bluegill Fry (BF-2) cells were purchased from the American Type Culture Collection (ATCC, Rockville, MD, USA; CCl-55/CRL2523/CRL-2872/ CCL-91, respectively). The cells were grown in Hyclone defined L-15 Leibovitz media supplemented with 10% fetal bovine serum (FBS) (Sigma-Aldrich, St. Louis, MO, USA) and 1% penicillin-streptomycin (Invitrogen, Thermo Fisher Scientific, Waltham, MA, USA) (complete L-15) at 20 °C with ambient CO_2_ (atmospheric air not supplemented with CO_2_). RTgill G3BP1 knockdown cells were maintained in the above-mentioned medium supplemented with 2 μg/mL puromycin as selectable marker.

### 2.2. VHSV Infection

EPC, RTG2 and RTgill cells were infected with indicated VHSV strain in serum-free L-15 at a multiplicity of infection (MOI) of 1. After 1.5 h, the virus-containing medium was removed, and cells were washed with phosphate-buffered saline (PBS) and replenished with complete L-15 media. In experiments involving inhibitors, cells were pretreated with inhibitors for 1 h prior to infection and added back post viral adsorption. Cycloheximide (CHX, 50 µg/mL) was added to the cell culture medium in some experiments to induce translation arrest.

### 2.3. VHSV Amplification and Titering

The recombinant Great Lakes VHSV strain (MIO3GL) were described previously [67]. VHSV Ia (F1 Strain) was provided by Dr. Gael Kurath (U.S. Geological Survey, Seattle, WA, USA). Recombinant virus stocks were prepared by infecting confluent EPC cells with a 1:1000 (*v*/*v*) dilution of unpurified virus stock from BF-2 infected cells in serum-free L-15. After 1.5 h, the virus-containing medium was replaced with complete L-15. Following the onset of cytopathicity (72–96 h), the virus-containing media and attached cells were subjected to a freeze–thaw cycle. Cellular debris was removed by centrifugation (6000 rpm, 20 min, 4 °C) and then clarified using a 0.22 µm syringe tip filter. Resulting supernatant was then subjected to ultracentrifugation through 25% Sucrose weight by volume (*w*/*v*) in TEN buffer (10 mM Tris HCl pH 7.5, 150 mM NaCl, 1 mM EDTA) at 25,000 rpm for 2 h at 4 °C. The virus-containing pellet was resuspended in phosphate-buffered saline (PBS) and stored at −80 °C until use.

Viral titer was determined by applying 1:10 serial dilutions in serum free L-15 of the purified recombinant virus to EPC cells. Following viral adsorption, a carboxymethyl-cellulose (CMC) overlay consisting of 0.5% CMC and 2% FBS was added. Following the onset of cytopathicity (72–96 h), infected cells were fixed with 10% formalin and stained with crystal violet. Viral plaques were counted and a final viral concentration (PFU/mL) were calculated for each viral grow up.

### 2.4. Chemicals, Plasmids and Antibodies

Cycloheximide and the PKR inhibitor C16 was purchased from Sigma-Aldrich (St. Louis, MO, USA); the PERK inhibitor GSK2656157 from Santa Cruz Biotechnology (Dallas, TX, USA); puromycin was from Fisher Scientific (Hampton, NH, USA). C16 (1 µM) and GSK2656157 (5 µM) were diluted in media prior to use and added.

EPC MAVS expression plasmid was obtained from Michel Bremont (French National Institute for Agricultural Research, Jouy-en-Josas, France). The IFNβ-luciferase reporter was obtained from John Hiscott (Insituto Pasteur Fondazione Cenci Bolognetti, Rome, Italy) and the MX1-luciferase reporter was described previously [69]. VHSV Ia N, G and NV target sequences were chemically synthesized and cloned into pcDNA3.1(-) (Invitrogen) using KpnI and HindIII restriction sites (BioBasic, Amherst, NY, USA). VHSV Ia P target sequence was PCR amplified and cloned into pcDNA3.1(-) using EcoRI and KpnI restriction sites. VHSV Ia M has been described previously [63]. Rainbow trout IFN (rtIFN) was obtained by UV-irradiating harvested infected media from RTG2 cells.

The antibodies to p-eIF2α (S51) (#3398) were purchased from Cell Signaling Technology (Danvers, MA, USA); eIF2S1 (#11170-1-AP) and β-actin (#66009-1-Ig) were purchased from Proteintech (Rosemont, IL, USA) and G3BP1 (TT-Y) (#sc-81940) was purchased from Santa Cruz Biotechnology (Santa Cruz, Dallas, TX, USA). Rabbit anti-VHSV was obtained from Vikram Vakharia (University of Maryland Baltimore County, Baltimore, MD, USA). All primary antibodies were used at 1:1000 in 5% (*w*/*v*) BSA/TBST. Anti-mouse IgG and anti-rabbit IgG HRP linked secondary antibodies were purchased from Cell Signaling Technology and ECL reagents were from Boston Bioproducts (Boston Bioproducts, Ashland, MA, USA). Alexa-488 and Alexa-647 conjugated anti-immunoglobulin antibody was purchased from ThermoFisher/Molecular Probes (Waltham, MA, USA). Antibodies against rainbow trout RIG-I, MDA-5, MAVS, PKR, PERK and IRF3 were generated based on expressed sequence tag (EST) sequences of *Onchorhynchus mykiss* from the NCBI database (https://www.ncbi.nlm.nih.gov/genbank, accessed on 19 November 2020.) based on homology with Human (*Homo sapiens)* proteins by Pacific Immunology (Ramona, CA, USA) and affinity purified using the antigen and used at 1:1000 dilution for immunoblots.

### 2.5. Cell Treatments

To induce heat shock stress, complete medium was prewarmed to 37 °C and added to cells cultured in standard conditions. The cells were then incubated at 37 °C for 1 h. To induce oxidative stress, cultured cells were treated with hydrogen peroxide (H_2_O_2_) at a final concentration of 5 mM for 3 h. Cells on coverslips were then analyzed by immunofluorescence microscopy and quantitated. To measure de novo protein synthesis, puromycin (10 µg/mL) was added to cells at indicated time points for 20 min prior to lysate collection and subject to immunoblot analysis.

### 2.6. Immunoblotting

Following indicated treatments, cells were lysed with NP-40 lysis buffer (0.5% NP-40, 90 mM KCl, 5 mM magnesium acetate, 20 mM Tris, pH 7.5, 5 mM β mercaptoethanol, 0.1 M phenylmethylsulfonyl fluoride (PMSF), 0.2 mM sodium orthovanadate, 50 mM NaF, 10 mM glycerophosphate, protease inhibitor (Roche Diagnostics, Indianapolis, IN, USA)). Samples were separated by SDS-PAGE and electrophoretically transferred to the nitrocellulose membrane (Biorad, Hercules, CA, USA). Membranes were blocked with 10% (*w*/*v*) nonfat dry milk/TBST for 2 h at room temperature. Primary antibodies were diluted in 5% (*w*/*v*) BSA/TBST (P-753, Boston Bioproducts, Ashland, MA, USA) and incubated overnight at 4 °C. The membrane was then incubated with a secondary antibody in TBST for 2 h at room temperature (Cell Signaling Technology, Danvers, MA, USA; Anti-mouse IgG, HRP-linked antibody #7076 and anti-rabbit IgG, HRP linked antibody #7074). Immunoreactive bands were visualized with ECL (Boston Bioproducts, Ashland, MA, USA) using a ChemiDoc-It^2^ 510 imager (UVP, Fisher Scientific; Hampton, NH, USA). For determining the ratios of p-eIF2α/T-eIF2α the intensity of each band was determined using Image J software (National Institutes of Health, Bethesda, MD, USA). Images were processed using Adobe Photoshop CS4 (Adobe, San Jose, CA, USA).

### 2.7. Immunofluorescence Microscopy

EPC, RTG2 and RTgill cells were seeded onto Poly-L-Lysine (0.01% *w*/*v*) coated coverslips (Sigma-Aldrich, St. Louis, MO, USA). Following indicated treatments, cells were fixed with 4% paraformaldehyde (BM-155; Boston Bioproducts, Ashland, MA, USA) for 15 min at room temperature and then permeabilized with 0.2% Triton X-100 in PBS for 15 min at room temperature. Cells were then blocked with 3% BSA, 0.02% Tween in PBS for 1 h at room temperature and then incubated the indicated antibodies at a 1:300 dilution in 3% BSA in PBS overnight at 4 °C. Cells were then incubated with a secondary antibody at a 1:300 dilution in PBS (Alexa 488- or Alexa 647-conjugated anti-immunoglobulin antibody, Molecular Probes; Eugene, OR, USA) for 1 h at room temperature. Cell nuclei were stained with DAPI (17985–50; EMS, Hatfield, PA, USA). Cells were imaged on an Olympus IX81 inverted fluorescence microscope and the analysis and processing of images were performed using Stream View and ImageJ software [70]. Cells containing G3BP1 puncta (*n* > 5) were considered stress granule positive and counted. The percentage of cells forming stress granules was calculated from at least 100 cells from various fields. Data are representative of three independent experiments.

### 2.8. Real-Time Quantitative PCR

RNA was isolated using TRIzol (Invitrogen, Thermo Fisher Scientific, Waltham, MA, USA) according to the manufacturer’s protocol. For reverse transcription (RT) reactions, Moloney Murine Leukemia Virus (M-MLV) reverse transcriptase (Promega, Madison, WI, USA) was used to reverse transcribe total RNA isolated. DNase I treatment was first performed by incubating RNA with 10X DNase buffer and 0.5 µL of DNase for 15 min at room temperature (Invitrogen, Thermo Fisher Scientific, Waltham, MA, USA). 1 µL of 25 mM Ethylenediaminetetraacetic acid (EDTA) was added to each reaction for 10 min at 65 °C to inactivate DNase. Reverse transcription reactions were carried out by incubating RNA with 100 ng of random hexamer primer and water at 70 °C for 10 min then briefly cooled on ice before addition of 5x MMLV RT buffer, MMLV RT, RNase inhibitor and dNTPs (Fisher Scientific, Hampton, NH, USA). Samples were incubated at 42 °C for 1 h followed by a 10 min incubation at 92 °C. Viral RNA samples were spiked with 1 ng of in vitro-transcribed green fluorescence protein (GFP) RNA to serve as an internal control for normalization. cDNA samples were diluted 1:10 with water and then quantified with quantitative reverse transcription PCR (RT-qPCR) using 5 µL of 2x SYBR qPCR Master Mix (1725120, Bio-Rad, Hercules, CA, USA), 2 µL of diluted cDNA, 50 ng of each primer and water to a total volume of 10 µL. Reactions and data collection were performed with a Bio-Rad C1000 real-time thermocycler for 3 min of initial denaturation at 95 °C, followed by 40 cycles of 15 s at 95 °C for denaturation and 30 s at 60 °C for elongation. Cycle threshold (Ct) values were obtained by an automated single point threshold within the log linear range and normalized to the spiked internal control GFP. Expression levels were calculated using the 2^−∆∆CT^ method. VHSV targeted primers were designed using GenBank accession GQ375941. Primer sequences used are listed in Table 1 below.

### 2.9. CRISPR Knockdown Cell Line

G3BP1 Knockdown cells were generated using the CRISPR/Cas9 system. Small guide RNA (sgRNA) was designed using GPP sgRNA Designer (https://portals.broadinstitute.org/gpp/public/analysis-tools/sgrna-design, accessed on 19 November 2020). The sequence used for generation of cell line is as follows; Forward: 5′CACCGccacaccaagatcagacatg3′ and Reverse: 5′aaaccatgtctgatcttggtgtggC3′. The guide RNA sequences were synthesized and cloned into the vector pSpCas9(BB)-2A-Puro (PX459; Addgene plasmid 62988) V2.0 as described previously [71,72]. In total, 20 μg of G3BP1 CRISPR plasmid was electroporated using Neon Transfection system (Invitrogen) in RTgill wild-type (WT) cells (1 × 10^6^, passage 10) as per manufacturer’s guidelines. Briefly, cells were pulsed once for 20 ms at 1400 V and recovered in growth medium. After 48 h, positive clones were selected using 2 μg/mL of puromycin containing L-15 medium and knockdown efficiency was verified by Immunoblotting.

### 2.10. Transfection

RTG2 cell transfections were performed using FuGENE (Promega, Madison, WI, USA) and RTgill transfections were performed using Viafect (Promega, Madison, WI, USA) according to manufacturer’s instructions. Plasmids were mixed with appropriate transfection reagent in serum-free L-15 at a 1:3 ratio for 10 min at room temperature and then added to cells in 500 µL of serum-free medium. Complete medium (1 mL) was added to cells after 3 h of incubation. Transfection remained at 20 °C for indicated time. VHSV Ia plasmids were electroporated in RTgill WT cells using Neon transfection system (Invitrogen). Briefly, cells (passage 10) were pulsed once for 20 ms at 1400 V and recovered in antibiotic free L-15 medium containing 10% FBS. After 24 h, media was replaced with L-15 medium containing 10% FBS and 1% penicillin-streptomycin.

### 2.11. Luciferase Assay

RTG2 and RTgill cells (3 × 10^4^) were plated in a 12-well dish and transfected 72 h post plating with indicated plasmids for 48 h, washed with PBS twice and then lysed for 15 min at 4 °C with 100 µL of 1X passive lysis buffer (Promega, Madison, WI, USA). In total, 50 µL of the clarified lysate was combined with 50 µL of luciferase buffer (250 mM Glycylglycine, 200 mM DTT, 100 mM ATP, 200 mM luciferin) and relative luminescence values determined using Molecular Device SpectroMax ID5 (Molecular Devices, LLC, San Jose, CA, USA). Luciferase values were normalized to total protein content of each sample determined by Bradford assay. Bradford assay was performed by adding 2 µL of lysate to 100 μL of 1X Bio-Rad Protein Assay Dye reagent concentrate (Bio-Rad, Hercules, CA, USA). Samples were briefly mixed and absorbance was read at 595 nm.

### 2.12. Virus Yield and IFN Bioassay

To assess viral replication and IFN secretion, media was harvested at indicated time points following infection of RTG2, RTgill WT or G3BP1 knockdown cells with VHSV Ia virus. Virus yield was determined by applying 1:10 serial dilutions in serum-free L-15 of harvested media to RTG2, RTgill WT or G3BP1 knockdown cells respectively. Following viral adsorption, a CMC overlay consisting of 0.5% CMC and 2% FBS was added. Following the onset of cytopathicity (72–96 h), infected cells were fixed with 10% formalin and stained with crystal violet (0.1% in methanol). Viral plaques were counted and a final viral concentration (PFU/mL) was calculated for each time point. IFN bioassays were used to assess IFN production during viral infection. Briefly, 1:3 dilutions of UV-irradiated medium from indicated samples were applied to RTgill WT or G3BP1 knockdown cells for 24 h. Following treatment with irradiated medium, cells were infected with VHSV IVb or VHSV Ia (MOI 0.1) for 72 h and then fixed with 50 µL of 100% methanol and stained with crystal violet (0.1% in methanol). One unit of IFN activity was determined by the dilution necessary to provide 50% protection from virus CPE.

### 2.13. Statistics

Data management, analysis and graphing were performed using the Prism7 software (GraphPad). Data were analyzed by two-tailed, unpaired Student *t* tests and are presented as the mean ± standard error of mean (SEM) or standard deviation (SD) as indicated. All data are representative of at least three independent experiments performed in triplicates.

## 3. Results

### 3.1. VHSV Infection Induces Stress Granule Formation

**Figure 1:**
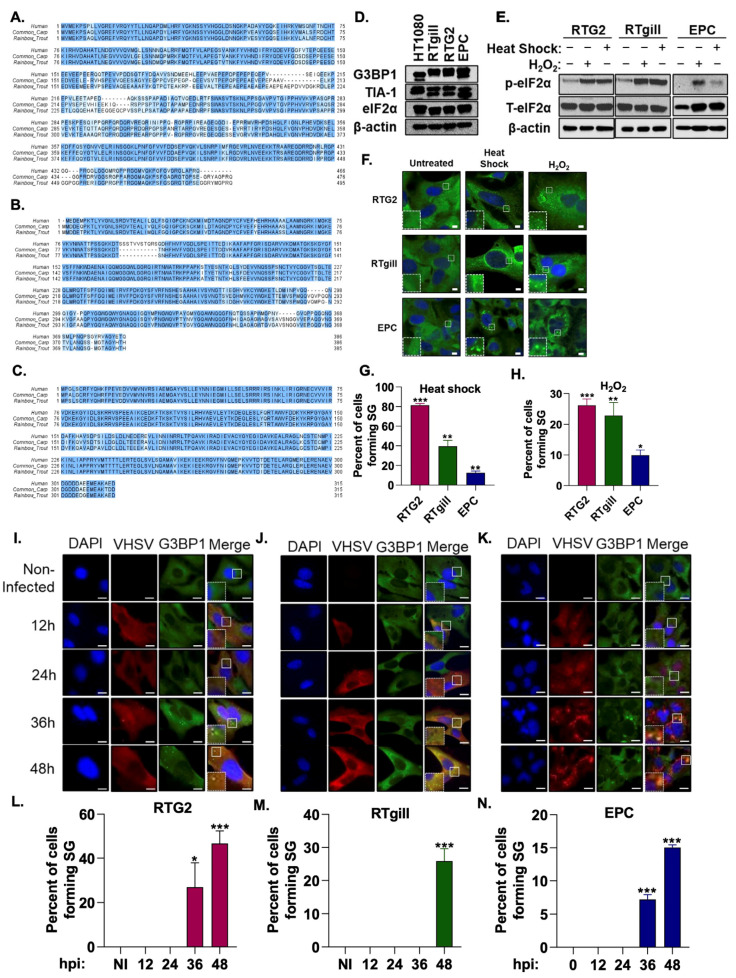
VHSV infection induces stress granule formation. Multiple amino acid sequence alignments of human, common carp and rainbow trout (**A**) G3BP1 (*Homo sapiens*, Q13283; *Cyprinus carpio*, XP_018929741; *Oncorhynchus mykiss*, XP_021417518;), (**B**) TIA-1 (*Homo sapiens*, P31483; *Cyprinus carpio*, KTF92398; *Oncorhynchus mykiss*, XP_021457804), (**C**) eIF2α (*Homo sapiens*, P05198; *Cyprinus carpio*, XP_018935983; *Oncorhynchus mykiss*, XP_021429557). Identical amino acids are highlighted with dark blue and similar amino acids are with light blue color. (**D**) Cell lysates of HT1080 (human fibrosarcoma), RTgill, RTG2 (rainbow trout) and EPC (Fathead minnow) cells were analyzed by immunoblotting with antibodies against G3BP1, TIA-1 and eIF2α. β-actin was used as loading control. RTG2, RTgill and EPC cells were subjected to heat shock (37 °C, 1 h), or oxidative stress (5 mM H_2_O_2_, 3 h) and (**E**) phosphorylation of eIF2α in cell lysates was analyzed by immunoblotting, or (**F**) cells were fixed and stained with anti-G3BP1 antibodies to monitor stress granules and DAPI to stain the nucleus and (**G**,**H**) percent cells forming stress granules were quantitated. RTG2 (**I**,**L**) and RTgill (**J**,**M**) cells were infected with VHSV Ia and EPC (**K**,**N**) cells were infected with VHSV IVb (MOI = 1 pfu/cell) at indicated times hpi cells were fixed and stained with G3BP1 and anti-VHSV antibodies. (L-N) Percent cells forming SG were quantitated. Data represents mean ± SD. Scale bar 1 μm; * *p* < 0.05; ** *p* < 0.01; *** *p* < 0.001.

Stress granules are produced during viral infection in mammalian cells but their role in fish cells remains poorly characterized. To characterize stress granule formation in fish, we first investigated if rainbow trout and common carp express proteins essential for stress granule formation. Sequence analysis identified homologs to the human proteins in ISR pathway including G3BP1 (65.8% identity with rainbow trout, 68.5% with carp), TIA-1 (81.3% identity with rainbow trout, 80.3% with carp), and eIF2α (90.7% identity with rainbow trout, 93.3% with carp) (Figure 1A–C). Cell lysates showed the expression of the ISR pathway and stress granule components in fish cells using human antibodies (Figure 1D). In mammalian cells, the phosphorylation of eIF2α occurs in the response to stressors such as heat shock, oxidative stress and viral infection to induce the formation of stress granules [33]. To determine if these stressors induce a similar response in fish cells, we incubated RTG2, RTgill and EPC cells at 37 °C for 1 h or in the presence of 5 mM H_2_O_2_ for 3 h. Like mammalian cells, increased levels of phosphorylated eIF2α in response to heat shock and oxidative stress was observed in cell lysates of RTgill, RTG2 and EPC (Figure 1E). We further investigated if phosphorylation of eIF2α by these stressors resulted in SG formation by visualizing the localization of a key stress granule protein, G3BP1 by immunofluorescence analysis. Compared to mock treated cells where G3BP1 showed diffuse cytoplasmic staining, heat shock and oxidative stress induced the formation of distinct puncta of stress granules in all cell lines. RTG2 and RTgill formed significantly more SG than EPC cells in response to heat shock and oxidative stress (Figure 1F–H). Taken together, these results suggest the conservation of the stress response pathway in fish.

To characterize the role of stress granules in fish, particularly in response to VHSV viral infection, we infected RTG2 and RTgill cells with VHSV Ia and EPC cells with VHSV IVb. We observed distinct G3BP1 puncta in the late stages of infection (36–48 h post infection (hpi)) in all three fish cell lines in contrast to the diffuse G3BP1 distribution in the non-infected cells (Figure 1I–N). These data suggest that both VHSV Ia and VHSV IVb induce the formation of stress granules during the course of infection.

### 3.2. Stress Granule Formation Requires PERK Activation

**Figure 2:**
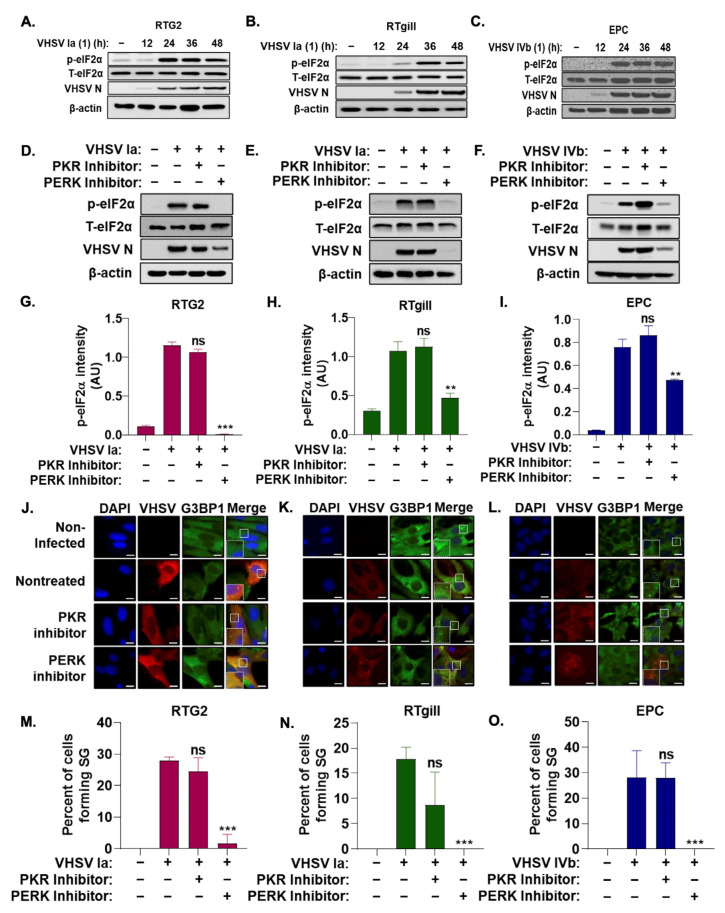
Stress granule induction during VHSV infection requires PERK activation. RTG2 (**A**), RTgill (**B**) were infected with VHSV Ia and EPC (**C**) with VHSV IVb (MOI = 1.0 pfu/cell) and cell lysates at indicated times was analyzed by immunoblotting with antibodies against, p-eIF2α, T-eIF2α, VHSV and β-actin. RTG2 (**D**), RTgill (**E**) and EPC (**F**) cells were treated with PERK inhibitor, or PKR inhibitor for 1 h prior to infection with VHSV Ia (RTG2, RTgill) or VHSV IVb (EPC) for 48 h and cell lysates analyzed by immunoblotting with antibodies against, p-eIF2α, T-eIF2α, VHSV and β-actin. (**G**–**I**) Quantification of (**D**–**F**). RTG2 (**J**,**M**); RTgill (**K**,**N**) and EPC (**L**,**O**) cells were treated with PERK or PKR inhibitor for 1 h prior to infection with VHSV Ia (RTG2, RTgill) or VHSV IVb (EPC) for 48 h and stress granule formation and infected cells were analyzed by staining with antibodies for G3BP1 or VHSV (**J**–**L**). Percent cells forming SG in (**J**–**L**) were quantitated (**M**–**O**). Data represents mean ± SD. Scale bar 1 μm; ns: not significant; ** *p* < 0.01; *** *p* < 0.001, ns = “not significant”.

The phosphorylation of eIF2α is a hallmark of the activation of the ISR, resulting in the formation of stress granules [1]. To monitor the impact of VHSV infection on the ISR, we used immunoblot analysis to monitor levels of phosphorylated eIF2α and viral protein levels during the course of VHSV Ia and VHSV IVb infection (Figure 2A–C). We observed a significant increase in levels of phosphorylated eIF2α by 24 hpi, but no significant change in levels of total eIF2α. The increase in phosphorylated eIF2α correlated with increased levels of viral proteins for both VHSV Ia and VHSV IVb, suggesting VHSV infection promotes phosphorylation of eIF2α as viral proteins are expressed.

Two hallmark serine/threonine kinases activated in response to viral infection that phosphorylate eIF2α and induce stress granules are PKR and PERK [9]. We investigated the role of PKR and PERK on the ISR and stress granule formation during VHSV infection by treating infected cells with or without inhibitors at concentrations previously determined in EPC cells, as indicated [68]. Western blot analysis demonstrated a significant decrease in levels of phosphorylated eIF2α when infected cells were treated with a PERK inhibitor compared to untreated VHSV-infected cells (Figure 2D–I). We also observed a significant decrease in viral protein expression with the addition of the PERK inhibitor. In contrast, PKR inhibitor during infection had no significant impact on levels of phosphorylated eIF2α or viral protein expression. These results suggest that the phosphorylation of eIF2α is mediated by PERK activation and the PERK-mediated phosphorylation of eIF2α during VHSV infection is required for viral replication. The treatment of VHSV infected cells with the PERK inhibitor resulted in a significant decrease in infected cells forming G3BP1 puncta compared to untreated but VHSV- infected cells as demonstrated by immunofluorescence (Figure 2J–O). PKR inhibitor had no significant impact on stress granule formation, further suggesting that PERK activation is required for the activation of the ISR and stress granule formation during VHSV infection.

**Figure 3:**
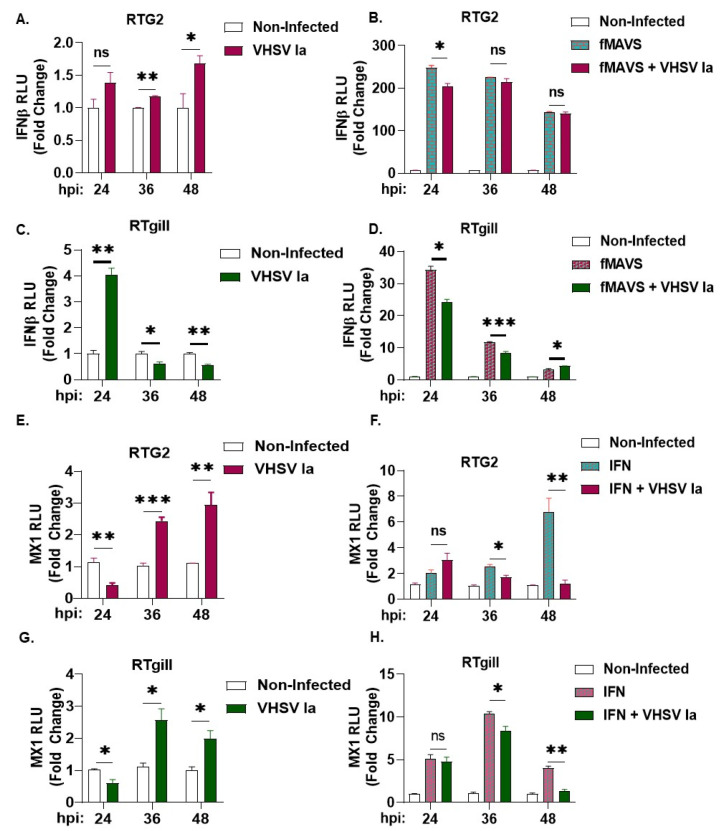
Regulation of host innate immune response by VHSV Ia. RTG2 cells (**A**,**B**) and RTgill cells (**C**,**D**) were transfected with IFN- β luc with or without fMAVS for 48 h, followed by infection with VHSV Ia for indicated times at MOI 1. Luciferase values were quantified and normalized to total protein levels and non-infected sample. RTG2 cells (**E**,**F**) and RTGill cells (**G**,**H**) were transfected with MX1-luc for 48 h, followed by infection with VHSV Ia for indicated times at MOI 1. Indicated cells were treated with rtIFN and luciferase values were quantified 24 h later and normalized to total protein levels and non-infected sample. Data represents mean ± SEM. ns, not significant; * *p* < 0.05; ** *p* < 0.01; *** *p* < 0.001, ns = “not significant”.

Virus infection in mammalian cells induces the formation of unique antiviral stress granules that play an essential role in antiviral signaling by regulating IFN production [33,34]. To determine the effect of VHSV infection on host response and IFN production in rainbow trout cells, we first assessed the impact of VHSV Ia infection on IFNβ promoter luciferase activity with and without the overexpression of fish MAVS (fMAVS). In RTG2 cells, with VHSV Ia infection alone, we observed an approximate 1.5-fold increase by 48 hpi, and in contrast a 4-fold increase was observed at 24 h in RTgill cells that decreased at later times of infection (Figure 3A,C). The ectopic overexpression of MAVS can induce IFN production and VHSV Ia infection inhibited fMAVS-induced IFN induction 24 hpi that was decreased at later times (Figure 3B,D). We further assessed the impact of VHSV Ia infection on IFN signaling by monitoring the effect of infection on the MX1, expression with and without exogenous IFN treatment. As expected, we observed a 3-fold increase in MX1 promoter activity in both RTG2 and RTgill cells with VHSV Ia infection alone (Figure 3E,G). However, treating cells with exogenous IFN and infecting with VHSV Ia, significantly reduced MX1 promoter activity in both RTgill and RTG2 cells compared to IFN treatment alone (Figure 3F,H). These data suggest that VHSV Ia induces IFN signaling upon viral infection in both RTG2 and RTgill cells, albeit with different kinetics. Further, these results suggest that VHSV can inhibit the ability of the cell to induce an antiviral response downstream of IFN production.

### 3.3. Role of Integrated Stress Response on IFN Signaling during VHSV Infection

**Figure 4:**
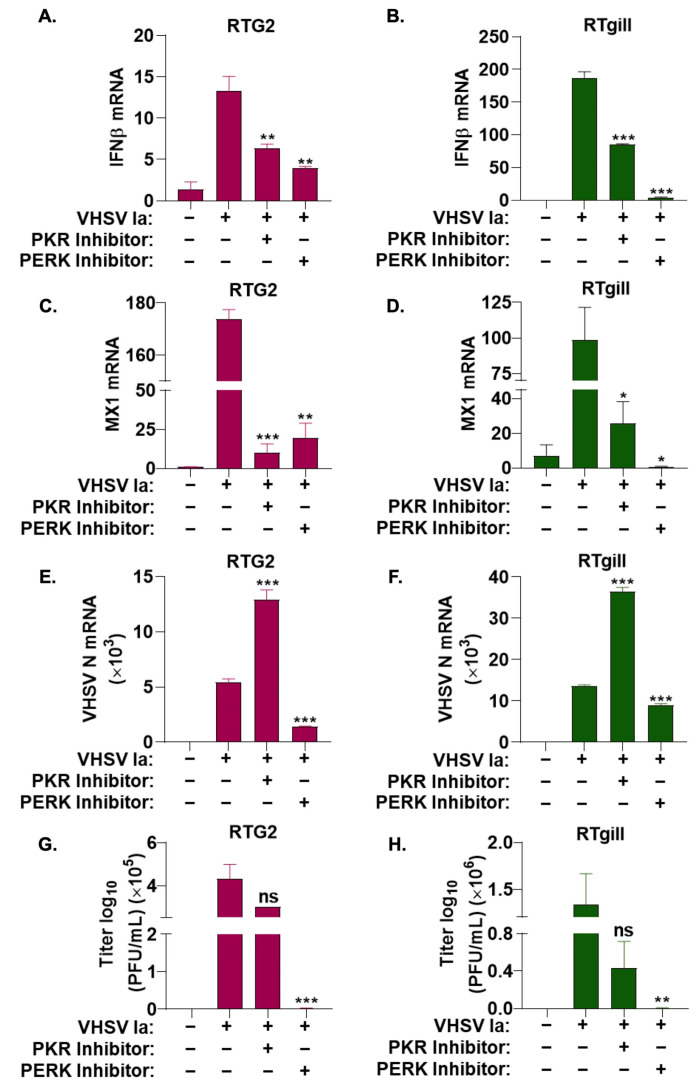
Role of ISR pathways on IFN signaling during VHSV infection. RTG2 (**A**,**C**,**E**,**G**) and RTgill (**B**,**D**,**F**,**H**) cells were treated with PERK inhibitor or PKR inhibitor 1 h prior to infection with VHSV Ia for 36 h and mRNA levels of (**A**,**B**) IFNβ, (**C**,**D**) MX1, or (**E**,**F**) VHSV N determined by RT-PCR. Data was normalized to spiked internal control and presented as fold change in expression compared to no treatment. (**G**,**H**) VHSV Ia titers were determined in supernatants of infected cells by plaque assays. Error bars reflect SEM. ns, not significant; * *p* < 0.05; ** *p* < 0.01; *** *p* < 0.001, ns = “not significant”.

We previously showed that VHSV IVb infection in EPC cells activated PERK to regulate IFN induction and host response [68]. To further investigate the role of the ISR and stress kinases on IFN signaling during VHSV Ia infection, we treated RTG2 and RTgill cells with either PKR or PERK inhibitor prior to VHSV Ia infection and assessed the mRNA levels of IFN and MX1 after 36 h. In both cell lines, compared to non-treated cells, we observed a significant decrease in levels of both IFN and MX1 mRNA with both PKR and PERK inhibitor treatments, PERK inhibitor strongly inhibited IFN and MX1 mRNA levels in RTgill compared to RTG2 cells. The inhibition of PKR had modest effect on mRNA levels of IFN and MX1 compared to PERK inhibition in both cell lines (Figure 4A–D).

These results suggest that while both PKR and PERK pathway are important for IFN production and signaling during VHSV Ia infection, PERK plays a more significant role.

To determine the impact of inhibition of the ISR pathway on VHSV Ia replication, RTG2 and RTgill cells were infected with VHSV Ia with or without PKR or PERK inhibitor treatment, and the 36 h later accumulation of viral mRNA levels was assessed. PERK inhibitor reduced levels of VHSV N mRNA by greater than 2-fold compared to untreated cells, while PKR inhibition increased VHSV N mRNA levels by 2.5-fold. Decrease in viral mRNA levels correlated with a corresponding decrease in viral titers in PERK inhibited RTG2 and RTgill cells, while PKR inhibition did not have any impact on viral titers compared to untreated cells (Figure 4E–H). Together, these results suggest that PERK is required for IFN production and signaling, which in turn affects viral replication.

### 3.4. Viral Replication Is Required for SG Formation

**Figure 5:**
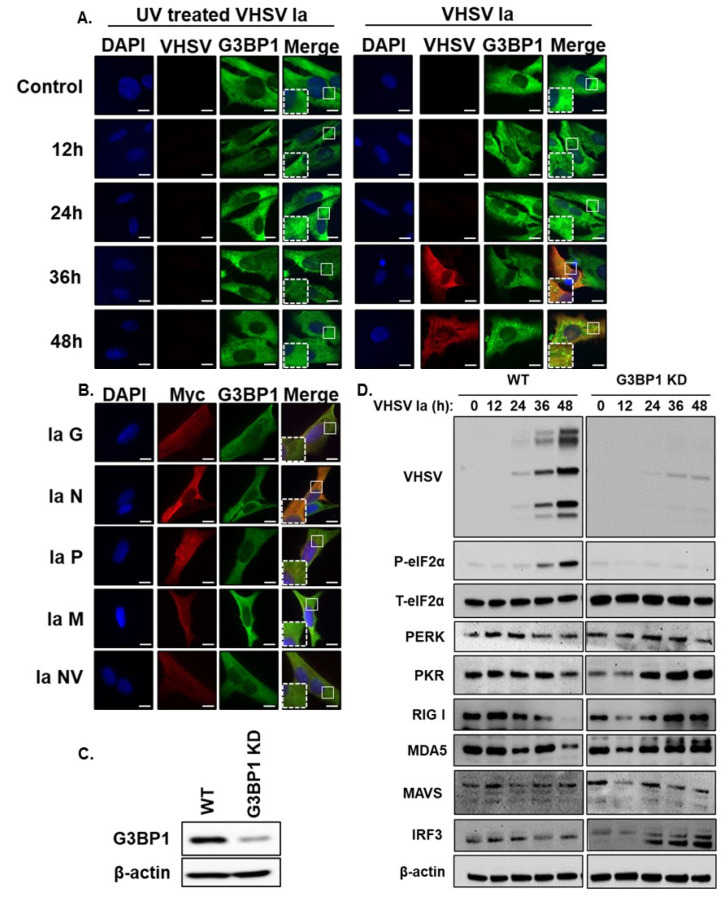
Viral replication is required for SG formation. (**A**) RTgill cells were infected with UV-inactivated or active VHSV Ia virus (MOI = 1) and after 48 h formation of stress granules was analyzed by staining with antibodies against G3BP1 and VHSV, (**B**) RTgill cells were transfected with myc-tagged plasmids for indicated VHSV proteins for 48 h and stress granule formation was analyzed by staining with antibodies against G3BP1 and myc, (**C**) CRISPR/Cas9 knockdown of G3BP1 in RTgill cells was verified by immunoblotting using G3BP1 antibody. β-actin was used as loading control. (**D**) WT and G3BP1KD cells were infected with VHSV Ia at MOI = 1 for indicated times. Cells lysates were separated and immunoblotted for indicated proteins. β-actin was used as loading control. Scale bar 1 μm; WT: wildtype; KD: knockdown; data are representative of results from two independent experiments.

Our results show that VHSV infection induces SG formation and requires the activity of PERK. To determine if VHSV replication and or any viral proteins produced during infection directly result in SG formation, we infected RTgill cells with UV-inactivated VHSV Ia that can infect cells but not replicate, and compared to cells infected with active replicating virus. Cells infected with active VHSV Ia formed G3BP1 puncta that correlate with the accumulation of viral proteins. The infection of cells with VHSV Ia incapable of replicating lacked G3BP1 puncta and as expected there was no accumulation of viral proteins (Figure 5A). To identify the viral proteins that may be involved in the formation of SGs during VHSV Ia infection, we transfected RTgill cells with plasmids expressing each of the individual VHSV structural and non-structural genes and after 48 h cells were analyzed for SG formation by staining with G3BP1 and myc antibodies. We observed no significant formation of G3BP1 puncta in cells overexpressing myc-tagged VHSV Ia viral proteins suggesting that no VHSV Ia protein alone is responsible for SG formation during VHSV Ia infection (Figure 5B). However, VHSV Ia replication intermediate is required for SG formation.

Stress granules induced in response to viral infection can be proviral or antiviral and G3BP1 is required for SG formation [20]. To investigate the role of G3BP1 during VHSV Ia infection, we generated the CRISPR-mediated knockdown of G3BP1 (G3BP1 KD) in RTgill cells and monitored the expression of phosphorylated eIF2α and the expression of viral proteins (Figure 5C,D). We observed a significantly decreased expression of all viral proteins in G3BP1 KD RTgill cells compared to WT RTgill cells, suggesting a proviral role of G3BP1 during VHSV Ia infection. Cells lacking G3BP1 showed a decrease in phosphorylated eIF2α implicating that G3BP1 is required for the phosphorylation of eIF2α during infection. To determine any effects G3BP1 may have on host antiviral proteins, we monitored the expression of components of IFN induction and signaling pathways in WT and G3BP1 KD cells after VHSV Ia infection. Compared to WT cells, in G3BP1 KD cells, we observed an increase in several ISGs that are induced by IFN including PKR, RIGI, MDA5 and MAVS, while IRF3 and PERK levels were relatively unchanged (Figure 5D). These results suggest that G3BP1 is important for inhibiting the antiviral response during VHSV infection, further suggesting a proviral role of G3BP1 during VHSV Ia infection.

### 3.5. G3BP1 Regulates IFN Signaling during VHSV Ia Infection

**Figure 6:**
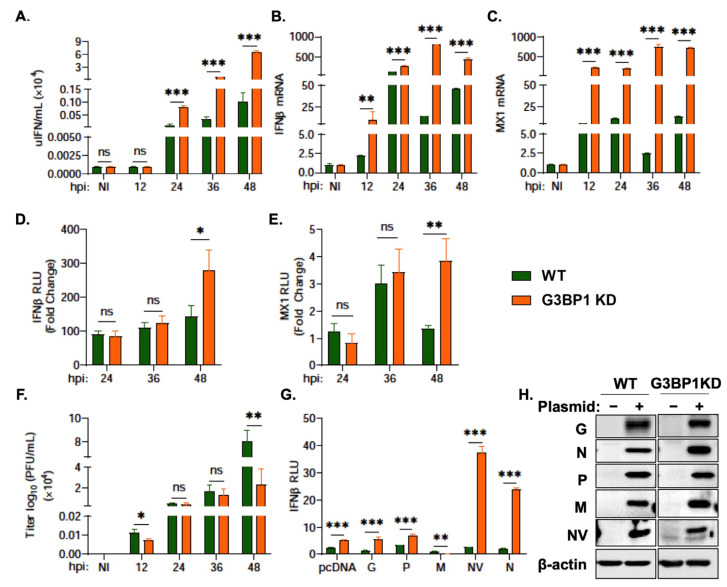
G3BP1 regulates IFN signaling during VHSV Ia infection. WT and G3BP1 KD cells were infected with VHSV Ia for indicated times at MOI = 1 and (**A**) media was collected at the indicated times post infection and used to quantitate IFN produced in IFN bioassays as described in methods, and mRNA levels of (**B**) IFNβ, (**C**) MX1 was measured by qRT-PCR and normalized to spiked internal control and presented as fold change in expression compared to no treatment. WT and G3BP1 KD cells were transfected with (**D**) IFNβ-luc or (**E**) MX1-luc for 48 h, followed by infection with VHSV Ia for indicated times at MOI = 1. Luciferase values were quantified and normalized to total protein concentration of each sample and non-infected sample. (**F**) WT and G3BP1 KD cells were infected with VHSV Ia for indicated times at MOI=1 and viral titers in supernatants was determined by plaque assay. (**G**) WT and G3BP1KD cell line were co-transfected with IFN luciferase, fish MAVS and myc-VHSV Ia proteins as indicated. pcDNA3.1 plasmid vector was used for transfection balancing and baseline control. Luciferase activity was analyzed at 72 h post transfection and RLU normalized to total protein concentration in each sample, (**H**) immunoblots comparing expression of myc-VHSV Ia proteins as indicated in cell lysates using myc antibodies and β-actin. The data represent Mean ± SD and was performed in triplicate. WT: wildtype; KD: knockdown; NI: not infected; ns, not significant; * *p* < 0.01; ** *p* < 0.001; *** *p* < 0.001, ns = “not significant”.

After observing an impact of G3BP1 KD on key antiviral proteins, mainly ISGs, we wanted to determine the impact of knocking down G3BP1 on IFN signaling. By 48 hpi, we observed significantly enhanced levels of IFN production (shown as units of IFN per mL) in G3BP1 KD cells (Figure 6A). A corresponding increase in IFN and MX1 mRNA levels was observed in G3BP1 KD cells infected with VHSV Ia (Figure 6B,C). The infection of IFNβ promoter or MX1 promoter luciferase transfected cells demonstrated a 3-fold and 4-fold increase, respectively, in both IFNβ and MX1 promoter activity with VHSV Ia infection in G3BP1 KD cells compared to WT RTgill cells (Figure 6D,E). As expected, the increased IFN levels in G3BP1 KD cells resulted in 4-fold reduced viral titers in culture supernatants of infected cells compared to WT cells after 48 h (Figure 6F). Taken together, these results further demonstrate that G3BP1 suppresses antiviral signaling during VHSV Ia infection. 

Previous studies have implicated several VHSV IVb genes in regulating IFN signaling. VHSV IVb M was shown to suppress host antiviral responses primarily at the transcriptional level and NV was shown to augment IFN signaling [63]. To identify the potential viral genes responsible for the impact of G3BP1 on IFN signaling, we co-transfected the plasmids expressing the individual VHSV Ia proteins with IFNβ promoter luciferase in WT RTgill and G3BP1 KD RTgill cells and measured IFNβ promoter activity induced by fMAVS overexpression (Figure 6G,H). VHSV Ia NV and N both demonstrated an approximate 40-fold and 25-fold increase, respectively, in IFNβ promoter activity in G3BP1 KD cells compared to WT RTgill cells. These results suggest a possible role of the NV and N in the ability of G3BP1 to shut down the host antiviral responses and IFN signaling.

### 3.6. G3BP1 Is Required for Viral Protein Expression

**Figure 7:**
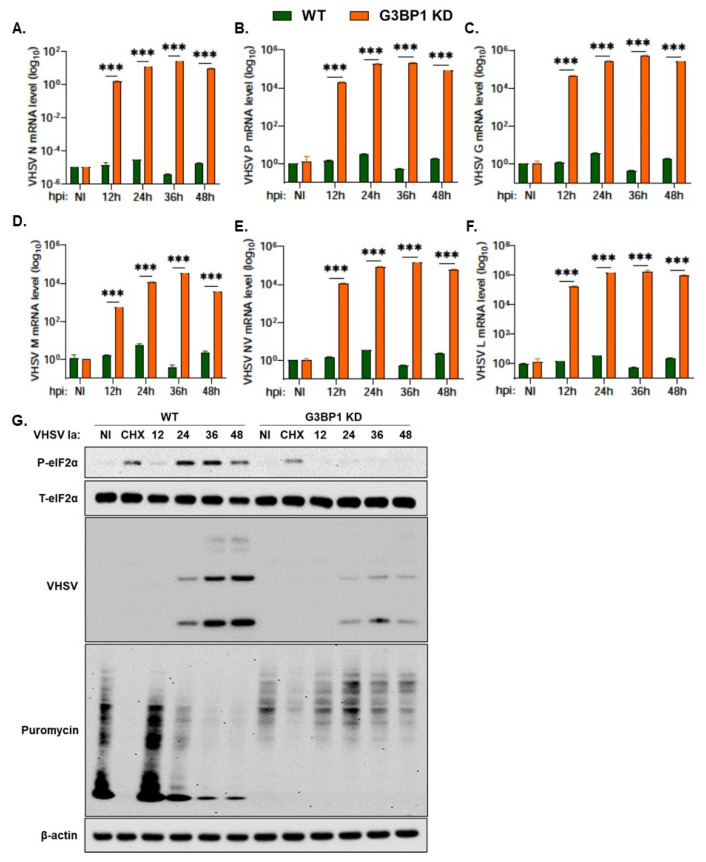
G3BP1 is required for viral protein expression. WT and G3BP1 KD cells were infected with VHSV Ia for indicated times at MOI = 1 and mRNA levels of (**A**) VHSV N, (**B**) VHSV P, (**C**) VHSV G, (**D**) VHSV M, (**E**) VHSV NV and (**F**) VHSV L was measured by qRT-PCR and normalized to spiked internal control and presented as fold change in expression compared to no treatment. (**G**) WT and G3BP1 KD cells were infected with VHSV Ia at MOI of 1. At indicated time points, cells were pulsed with puromycin (10 µg/mL) for 20 min and cell lysates were analyzed by immunoblotting using antibodies against p-eIF2α, T-eIF2α, puromycin and viral proteins were detected using VHSV antibodies. β-actin was used as loading control. Data are representative from two individual experiments. The data represent mean ± SD; WT: wildtype; KD: knockdown; NI: not infected; CHX: cycloheximide. *** *p* < 0.001.

Our results show an important role of G3BP1 in regulating IFN production during VHSV Ia infection and cells lacking G3BP1 produced higher levels of IFN and reduced viral titers. To determine if G3BP1 had any direct impact on viral replication, we monitored levels of viral mRNA during a time course infection in G3BP1 KD cells and compared to WT RTgill cells. Despite previously observing a decrease in viral protein expression level in infected cells with the knockdown of G3BP1, we observed a significant increase in levels of viral mRNA with the KD of G3BP1 (Figure 7A–F). Interestingly, despite the significant increase in levels of viral mRNA in G3BP1 KD cells, we observed a 0.6 log decrease in viral yield in G3BP1 KD compared to WT RTgill cells (Figure 6F). These results suggest that G3BP1 may play an important role in the synthesis of viral proteins, in addition to the impact on IFN signaling.

The stress response pathway activated in response to viral infection results in the shutoff of host protein translation via phosphorylation of eIF2α. To determine the impact of knocking down G3BP1 on the shutoff of host translation during VHSV infection, we pulsed infected cells at the indicated time points with puromycin for 20 min prior to making cell lysates (Figure 7G). Puromycin incorporates into newly translated polypeptides and terminates translation. We can therefore use immunoblot analysis and an anti-puromycin antibody to monitor newly synthesized proteins. As previously shown, we observed a decrease in levels of phosphorylated eIF2α and viral proteins in G3BP1 KD cells compared to WT RTgill cells (Figure 7G). In WT RTgill cells, we observed a decrease in puromycin incorporation as VHSV infection progresses to 48 h demonstrating that VHSV infection shuts down host protein translation. The KD of G3BP1 during VHSV infection resulted in increased levels of puromycin incorporation at the same time points (Figure 7G). These results suggest that G3BP1 is essential for the shut off of host protein translation during VHSV Ia infection. Cells lacking G3BP1 show reduced viral protein accumulation; however, host translation is rescued.

## 4. Discussion

In response to different stress stimuli, such as viral infection, oxidative stress and heat shock, eukaryotic cells activate the ISR to attempt to restore cell homeostasis. The converging point of the ISR is the phosphorylation of eIF2α, which results in the inhibition of cap dependent translation. The accumulation of stalled translation initiation complexes and RNA-protein interactions results in the liquid-liquid phase separation of the aggregates from the rest of the cytoplasm leading to stress granule formation. Viral-induced stress granules, referred to as antiviral stress granules, have been proposed to provide a platform for the efficient interaction of RNA ligands with receptors and play a role in antiviral signaling. However, viruses have developed mechanisms to promote survival by inhibiting stress granule formation, while some viruses have developed mechanisms to manipulate the stress granule formation to benefit viral replication. Our results show that fish cells, like mammalian cells, express proteins in the ISR pathway including G3BP1, TIA-1 and eIF2α and formed SG in response to heat shock and oxidative stress. Previous studies showed the VHSV IVb infection of fish cells activated stress kinase PERK, resulting in eIF2α phosphorylation and host translation shut-off [68]. This led us to explore SG formation in response to VHSV infection and its impact on host response and viral pathogenesis. We show that VHSV infection in RTG2, RTgill and EPC cells activates ISR pathway causing eIF2α phosphorylation and SG formation. Among the stress kinases, PERK is specifically required for SG formation as PERK inhibitor reduced eIF2α phosphorylation and viral protein expression with a concomitant decrease in viral titers, while PKR was not required for this effect. However, both PERK and PKR are important for IFN production and signaling. Next, we demonstrated that SG formation during VHSV required viral replication and the expression of individual viral proteins was not sufficient to induce SG formation. Finally, utilizing CRISPR/Cas9 knockdown of G3BP1 in RTgill cells, we demonstrated a decrease in VHSV viral titer and an increase in immune signaling. We propose that during VHSV infection, the activation of the ISR pathway and the formation of SG plays a critical role in VHSV pathogenesis with a direct role of G3BP1 in regulating viral and host protein expression.

The activation of the stress response pathway and SG formation during virus infection is well characterized in mammalian cells, unlike lower vertebrates. A homology search analysis of key proteins of the ISR pathway revealed the significant homology of G3BP1, TIA-1 and eIF2α between human, rainbow trout and carp and human antibodies detected the expressed proteins in cell lysates (Figure 1A–E). Similar to mammalian cells, SG formation in response to diverse stimuli such as heat shock, oxidative stress and virus infection was detected in fish cells by visualizing the formation of G3BP1 puncta suggesting conserved pathways (Figure 1F–N). The response to the stressors, determined by the number of SG formed, varies among the fish cells analyzed suggesting differences in mechanisms between cell lines and the response of cells to different viral strains.

Among the ISR kinases, PERK and PKR are activated in response to virus infection and phosphorylate eIF2α resulting in translation arrest, a key step in SG formation. Our results show that VHSV Ia infection, similar to previous observations made during VHSV IVb infection, induced the phosphorylation of eIF2α and levels of phosphorylated eIF2α increased as viral infection progressed (Figure 2A–C) [68]. The inhibition of PERK, and not PKR, inhibited p-eIF2α accumulation with a corresponding decrease in viral protein expression and SG formation suggesting PERK/eIF2α axis activated by VHSV can regulate viral pathogenesis through an effect on the translation of viral proteins and SG (Figure 2D–O). SG formed during viral infection, avSG, are unique and harbor antiviral proteins to regulate innate immune response and IFN production [33,34]. To determine the effect of SG in VHSV Ia infection, we first compared IFN production and the activation of the IFN-stimulated gene, MX1, in VHSV Ia infected in rainbow trout cells. Compared to RTG2 cells, RTgill cells induced IFN early followed by MX1 induction and shut down during later stages of infection (Figure 3A,C,E,G). Similar to VHSV IVb, Ia strain shuts down IFN and MX1 induction in cells over-expressing MAVS or treated with exogenous IFN prior to infection (Figure 3B,D,F,H). In our experiments, we infected with VHSV 48 h post MAVS transfection, a timing geared towards allowing replication to occur in concert with the upregulation of IFN expression to avoid the complete suppression of virus infection or replication. While the amount of MAVS expressed likely had an impact on VHSV replication overall, our data show that enough virus escaped to impact IFN upregulation, and this effect was more significant in RTgill cells compared to RTG2 cells. The results shown here suggests the virus “fighting back” as has been previously reported during VHSV IVb infection in EPC cells pretreated with IFN prior to infection [63]. A similar impact on MX1 expression following IFN production by fMAVS has been demonstrated by expressing individual Infectious Hematopoietic Necrosis Virus (IHNV) genes in various fish cell lines [73]. These results support the notion that VHSV can inhibit host antiviral responses at later stages and suggests a cell type specificity in the impact of VHSV Ia on the host cell response. It is important to note that the gill epithelium is the site of infection for VHSV and therefore, the virus may be more efficient at shutting down the host cell response in the gill cells to facilitate infection in the wild [74].

SG formation by VHSV is inhibited when PERK activity is inhibited. To correlate antiviral response with SG formation, we compared IFN induction with the accumulation of viral proteins and its impact on VHSV replication when PERK/eIF2α axis or PKR was inhibited. With the inhibition of PERK, we observed a significant decrease in both innate immune signaling genes (IFN and MX1) and a significant decrease in both viral RNA levels and viral yield, compared to nontreated infected cells (Figure 4). These results suggest that the activation of the ISR and stress granule formation are required for not only efficient viral replication, but also plays an important role in modulating the innate immune response. The decrease in stress granule formation with the inhibition of PERK, as well as the significant decrease in viral yield, suggests that VHSV Ia infection utilizes PERK induced stress granules in a proviral manner. Depending on the biological properties of the viruses, the differential modulation of PERK activity has been observed in mammalian cells. During Vesicular Stomatitis Virus (VSV) and Hepatitis C Virus (HCV) infection, the specific activation of PERK favors viral replication by degrading type I IFN receptor, IFNAR1, and suppressing IFN signaling and antiviral effect [75]. Bovine Viral Diarrhea Virus activates PERK to increase viral gene products during infection by affecting translation [76]. Inhibiting unfolded protein response pathway (UPR) to alleviate ER stress inhibited Murine Coronavirus (MHV) and Severe Acute Respiratory Syndrome-associated Coronavirus 2 (SARS-CoV2), suggesting CoV subvert UPR to facilitate replication [77]. In contrast, West Nile Virus (WNV) and Langat Virus replication is inhibited by PERK signaling [78,79]. Previously, we showed a role for the non-virion protein NV of VHSV IVb in VHSV pathogenesis by utilizing the PERK-eIF2α pathway for viral mediated host shutoff and IFN signaling to regulate host cell response, while promoting viral protein synthesis [68]. Interestingly, we also observed a significant decrease in immune signaling genes (IFN and MX1) with a PKR inhibitor, despite previously demonstrating no significant impact of PKR on stress granule formation and the activation of the ISR (Figure 4A–D). This suggests that VHSV may regulate innate immune signaling during viral infection through multiple mechanisms. We also demonstrated a significant increase in levels of viral mRNA (Figure 4E,F) with the PKR inhibitor, despite observing no significant impact on viral yield (Figure 4G,H) or viral protein expression (Figure 2D–I). It is likely that similar to VSV, during VHSV infection, translation arrest or the binding of PKR to dsRNA, viral replication intermediates may prevent the availability of viral proteins required for viral genome replication and the amplification of mRNA transcription in infected cells [18]. Therefore, inhibiting PKR may result in reduced IFN production and the increased availability of viral mRNA. Other studies have demonstrated crosstalk between PERK and PKR during VSV infection and additional studies may be needed to address if similar mechanism occurs in VHSV-infected cells [80]. Together, these observations suggest that PERK activity is required for host translation shut-off and efficient viral protein synthesis in VHSV Ia infection. However, its direct impact on viral replication requires further investigation in future studies.

VHSV Ia infection caused significant increase in eIF2α phosphorylation, host translation arrest and SG formation, however viral proteins continue to be synthesized. Similar observations were made in response to VHSV IVb infection in EPC cells [68]. We first sought to clarify whether any VHSV products, viral proteins or replication intermediates, act to promote SG formation. The infection of RTgill cells with UV-inactivated virus that is unable to replicate in cells failed to induce SG compared to robust SG formation with active VHSV Ia replication, demonstrating the need for a replication intermediate in SG induction (Figure 6A). One of the common mechanisms employed by viruses is to interfere or promote SG formation by targeting eIF2α phosphorylation or use viral proteins to sequester SG components and inhibit SG assembly, or shield viral components from host detection in SG to promote viral replication. For instance, Hepatitis C Virus co-opts G3BP1 protein to regulate genome replication, viral assembly and egress [41,42]. Ebola Virus (EBOV) encodes VP35 that inhibits SG assembly by sequestering G3BP1 in viral inclusion bodies to favor replication, suggesting an important role of SG in viral pathogenesis [81]. HSV-1 expresses vhs protein that inhibits the phosphorylation of eIF2α [82]. Our results show that the individual expression of the VHSV Ia-encoded structural or nonstructural proteins alone cannot induce SG (Figure 5B). While not explored, it is possible that multiple viral proteins may together mediate this effect as it happens in a virally infected cell. Based on our previous studies with VHSV IVb, we speculate NV protein is required for the PERK mediated p-eIF2α and efficient viral protein synthesis with possible role in SG formation [68].

G3BP1 is a critical regulator of SG dynamics, RNA turnover, and translation. More recently, its role as a regulator of immune response highlights an important role in the crosstalk of virus-induced stress response and the activation of IFN signaling pathway [36]. SG formed during virus infection, avSG, recruit antiviral proteins and G3BP1 is required for its assembly [20,33,34]. We generated G3BP1 knockdown in RTgill cells using CRISPR/Cas9 gene editing (Figure 5C) and compared ISR activation and host innate immune response in cells infected with VHSV Ia. In cells with G3BP1 knockdown (G3BP1 KD), surprisingly, eIF2α was very weakly phosphorylated and a very low accumulation of viral proteins was detected compared to wild-type (WT) cells. No significant increase in total eIF2α or PERK, its upstream kinase, was detected on immunoblots. However, our results show that protein levels of ISGs such as PKR, RIG-I, MDA5 and MAVS increased at later infection times in G3BP1 KD cells. The expression of IRF3, a transcription factor required to induce IFN, did not vary between WT and G3BP1 KD cells (Figure 5D). These results suggest that G3BP1 is required for both host and viral protein translation, and in the context of viral infection, immune signaling proteins like IFN. As expected, G3BP1 KD cells produced significantly higher amounts of IFN measured in culture supernatants, that correlated with increased IFN mRNA levels and IFN promoter activity. An increase in IFN production induced higher levels of MX1, an ISG, as is evident by an increase in mRNA level and MX1 promoter activity (Figure 6A–E). Predictably, viral titers were low in G3BP1 KD cells, demonstrating a proviral effect of G3BP1. A recent study in another fish model of Grouper spleen cells infected with Red-Spotted Grouper nervous necrosis showed the requirement of G3BP1 and SG formation with an antiviral effect highlighting the diverse roles of G3BP1 and SG in viral infections [83]. In line with our observations, another study showed that G3BP1 is critical for murine and human norovirus replication. The authors showed that G3BP1 recruits ribosomal subunits and facilitates the translation of viral RNA using viral protein genome-linked (VPg) attached to the 5′ end of RNA genome [45]. Furthermore, the redistribution of G3BP1 to viral replication complexes promotes efficient viral replication [44]. Since G3BP1 KD cells produced significantly higher IFN, we sought to identify if any VHSV Ia proteins may augment IFN production in G3BP1 KD cells. The expression of VHSV Ia NV protein in RTgill cells upregulated IFN promoter activity 38-fold over WT cells and Ia N protein caused 25-fold increase (Figure 6G). Based on our previous study on VHSV IVb NV protein in host translation shut-off via PERK, and our observations on IFN induction in G3BP1 KD cells, we speculate that G3BP1 may directly or indirectly regulate viral translation by interacting with NV protein, while the contribution of N protein cannot be ruled out. Future studies will address if Ia NV and or N protein bind G3BP1 and if RNA-binding properties of G3BP1 is required for regulating translation as well as SG assembly.

G3BP1 KD cells produced less virus compared to WT cells and lesser viral proteins accumulated during the time course of VHSV Ia infection (Figure 5D and Figure 6). Unexpectedly, levels of all viral mRNA were significantly high in G3BP1 KD cells, suggesting a global effect as IFN and MX1 mRNA levels were also high (Figure 7A–F). Despite significant increases in viral mRNA levels, no increase in viral protein expression is observed with corresponding reduction in viral yield (Figure 5D and Figure 6). To determine the broader effect of G3BP1 on host translation shut-off, the incorporation of puromycin into newly translated proteins in VHSV Ia infected cells was analyzed in WT and G3BP1 KD cells. Infection with Ia virus causes significant decrease in puromycin incorporation into cellular proteins, however viral proteins continue to be synthesized (Figure 7G). In contrast, in G3BP1 KD cells, viral protein synthesis is significantly reduced, while cellular proteins continue to be synthesized. As our data suggests that G3BP1 is required for the translation of viral mRNA, the observed increase in viral mRNA could be due to the accumulation of viral mRNA in the absence of protein synthesis, coupled with the broader effect of the lack of turnover in SG. It is also likely that VHSV may utilize a virally encoded protein, possibly NV, to recruit G3BP1 and ribosomes in a cap-independent manner to initiate translation on viral mRNAs. In that context, Vesicular Stomatitis Virus (VSV), requires RPL40 for VSV mRNA translation, but RPL40 is not required for general cap-dependent protein synthesis of host proteins [84]. It is conceivable that viruses may use specific host proteins to regulate translation of viral proteins rather than broad shut down through eIF2α activity to provide selective advantage when host translation is arrested.

Our study suggests that VHSV uses diverse mechanisms that work cooperatively to regulate innate immune response through host gene expression and controlling host translation to promote viral replication. The balance of these processes dictates the outcome of infection. VHSV induces SG formation and in the early stages it may be used to produce IFN and as viral infection proceeds the expression of viral proteins and the redistribution of SG protein, G3BP1, for effective viral mRNA translation may tip the translation in favor of viral proteins. In the absence of G3BP1, viral mRNA levels increase possibly due to a decrease in viral protein synthesis, or a decrease in RNA turnover or both. Our results also suggest that SG formation and host translation arrest may be uncoupled and future studies will allow the detailed characterization of the two events in VHSV pathogenesis. We demonstrate a novel role of G3BP1, in addition to SG formation, as a host proviral factor required for viral translation and suggests G3BP1 can be used as a therapeutic target to control VHSV replication.

## 5. Conclusions

Fish cells respond to stress stimuli including virus infection by inducing stress granules. The infection of rainbow trout cells, RTG2 and RTgill with VHSV Ia virus activates ER-stress kinase, PERK, and induces stress granules. Using inhibitors, we show that the activity of PERK is required for stress granule induction, IFN production, antiviral signaling and viral replication. G3BP1 depleted RTgill cells produced significantly higher amounts of IFN, expressed higher antiviral genes and accumulated viral mRNA, while viral protein expression was impaired resulting in reduced viral titers. Our studies identify an important role of ISR activation and stress granule formation and a previously unrecognized proviral role of G3BP1 in VHSV pathogenesis.

## Figures and Tables

**Table 1:** Primers for qRT-PCR.

Primer	Sequence (5′-3′)
GFP se	ATGGTGAGCAAGGGCGAGGA
GFP as	TAGCGGCTGAAGCACTGCACGCC
VHSV Ia N qRT se	TTGATGAGACAGGTGTCAGAGG
VHSV Ia N qRT as	TTGGAGTTGTCATTGAGTCCAT
VHSV Ia P qRT se	GCTCCTGAGACGTATCAAGATG
VHSV Ia P qRT as	CATTTTCCTTTTGAGACTCCAG
VHSV Ia G qRT se	GGTGACTGTGACTATGAGGCAG
VHSV Ia G qRT as	CAACTTGTCCCCAAATATCATG
VHSV Ia M qRT se	TATGATCTTTGGAGAAACCAGC
VHSV Ia M qRT as	GTCACACTCCCATGTCTAATGG
VHSV Ia NV qRT se	GCGAGATGATCACACACAGACT
VHSV Ia NV qRT as	CCCTCAGATCATCTAGGATCCT
VHSV Ia L qRT se	AGAGAGCACATCAGGTACCAAG
VHSV Ia L qRT as	GCTCTGTGTCTTCAAAAGATGG
Fish IFN se	GATGCTGAGTTTGAGGACAAAGTC
Fish IFN as	GTTTCATGGCAGGTGATACACAGGA
Fish MX-1 se	ATTAACCTGGTTGTGGTGCCATGC
Fish MX-1 as	TACCACTGTCCCTTCAGTGCCTTT

## Data Availability

All data relevant to this study can be found in this article.
